# BARRIERS TO HEPATITIS C TREATMENT AMONG US BABY BOOMERS: PROVIDER PERSPECTIVES

**DOI:** 10.1093/geroni/igad104.2577

**Published:** 2023-12-21

**Authors:** Elaine Wethington

**Affiliations:** Cornell University, Ithaca, New York, United States

## Abstract

Chronic Hepatitis C (HCV) is a major health problem in the US for the baby boomer cohort (born 1945-65), affecting 1.8 million persons; 75% acquired it through injection drug use in earlier life. About 20% develop cirrhosis, often leading to end stage liver disease. HCV is curable and treatment has become more affordable and available. However, uptake of treatment has lagged. This paper reports on a semi-structured interview study of 36 health providers from 2 states (NY, AL) with experience treating/referring HCV+ patients, part of a mixed-methods study of providers and patients. Interviews were coded using thematic analysis. Providers reported that baby boomers, especially racial/ethnic minority and low income, experience barriers to HCV care, because of the complexity of the treatment cascade (testing, linkage to qualified providers, prescribing treatment, insurance delays, and successful completion of therapy). Low provider awareness of need to test for and treat HCV, limited access to specialists (the primary prescribers of HCV medications until recently), variable effectiveness of health systems in assuring successful treatment, and insurer initial reluctance to support treating all infected persons, have also contributed to disparities in care. Perceived stigma and discrimination are barriers to treatment even among the majority of newly diagnosed HCV+ baby boomers who no longer use injection drugs. Providers report that patients fear that families and workplaces will avoid or isolate them if their past drug use becomes known. Moreover, patients who have yet to experience serious health impact may delay treatment, given fears about discrimination against drug users.

